# Asymmetric lung increases particle filtration by deposition

**DOI:** 10.1038/s41598-023-36176-3

**Published:** 2023-06-03

**Authors:** Debjit Kundu, Mahesh V. Panchagnula

**Affiliations:** Department of Applied Mechanics, Indian Institute of Technology Madras, Chennai, Tamil Nadu 600036 India

**Keywords:** Biomedical engineering, Respiration, Fluid dynamics

## Abstract

Human lung is known to be an asymmetric dichotomously branched network of bronchioles. Existing literature on the relation between anatomy and air-flow physics in the tracheobronchial trees has discussed the results of asymmetry. We discuss a secondary (but an important) lung function to seek asymmetry: to protect the acinus from a high pathogen load. We build morphometric parameter-based mathematical models of realistic bronchial trees to explore the structure-function relationship. We observe that maximum surface area for gas exchange, minimum resistance and minimum volume are obtained near the symmetry condition. In contrast, we show that deposition of inhaled foreign particles in the non-terminal airways is enhanced by asymmetry. We show from our model, that the optimal value of asymmetry for maximum particle filtration is within 10% of the experimentally measured value in human lungs. This structural trait of the lung aids in self-defence of the host against pathogen laden aerosols. We explain how natural asymmetric design of typical human lungs makes a sacrifice away from gas exchange optimality to gain this protection. In a typical human lung, when compared to most optimal condition (which is associated with symmetric branching), the fluidic resistance is 14% greater, the gas exchange surface area is about 11% lower, the lung volume is about 13% greater to gain an increase of 4.4% protection against foreign particles. This afforded protection is also robust to minor variations in branching ratio or variation in ventilation, which are both crucial to survival.

## Introduction

The primary function of the lung is to facilitate gas exchange between atmospheric air and the blood in the acinus. Here, gas exchange takes place across an extremely thin membrane ($0.6 \,\upmu \textrm{m}$) over a large surface area ($70 \,\textrm{m}^2$)^1^. The mechanisms of airflow through the airways and gas diffusion across the alveolar membrane have been extensively studied by both the medical and fluid mechanics communities. A secondary (equally important but less studied) purpose served by the lung is to ensure that the air that reaches the acinus, is relatively pathogen-free. The inhaled air could contain several impurities—smoke, dust, pathogen carrying droplets as well as harmful toxins in the form of aerosols, which may lead to multiple health hazards if they reach the deep lung. As this particle-laden air passes through the airways, particles largely deposit along the mucousal lining of the airways. The branched structure of the airways also serves as a mechanical filter for these particles.

Among the various organ systems of the body, the digestive and the pulmonary systems are most exposed to a high load of foreign material through ingestion of food and inhalation of air, respectively. While the gastrointestinal tract has a bio-chemical defence mechanism of its own, the defence mechanism of the pulmonary system, is mostly physical in origin. This is because the body has no mechanism of cleansing pathogens suspended in air without them first depositing on the walls. The lungs are indeed equipped with an immunological defence mechanism, but can only be activated after particles are deposited on the tracheobronchial walls.

In this manuscript, we will argue that asymmetry in lung morphology will afford one such protection mechanism for the body. Lung branching asymmetry is widely acknowledged but its evolutionary motives are poorly understood. At first glance, it appears that lung asymmetry results in a reduction in alveolar surface area, increased breathing work, and greater overall volume occupied by the airway tree—all leading to degradation in lung function. Therefore, it may seem that asymmetry hampers the optimal design of the lungs. However, we demonstrate that asymmetric airway branching optimizes a lesser-discussed secondary function of the lung—aerosol filtration - while sacrificing some of the primary functional efficiency.

It is well known that the airways within human lungs form a tree-like structure with repetitive dichotomous branching. Air enters the body through either the nose or mouth and passes through the glottis into the trachea. The singular trachea bifurcates into two main bronchi connecting into each of the two lungs. They subsequently branch into two secondary bronchi each; each of which subsequently go on dividing dichotomously into four, eight, and so on into smaller bronchi, termed as bronchioles. Each level of division is termed a branching *generation*. The bronchioles divide multiple times (approximately 23 generations^2^) before terminating into alveolar sacs. The geometry is often simplistically modeled as a self-similar^3^, space-filling fractal tree formed by cylindrical tubes. A striking feature of lung geometry is its asymmetry—the right lung is larger in size than the left lung. Although the main reason for the left lung being smaller is to accommodate the heart, this asymmetry appears to propagate down to the airway bifurcation units as well, where parent branches bifurcate into daughter branches of unequal dimensions. As Miguel^4^ in his recent work remarks, the functional importance of asymmetry on airflow design or on the possible effect it may have on the optimality due to deviation from symmetry has not been discussed in the literature. That forms the core motivation of this work: to explore optimality arising out of deviation from symmetry by treating the lung as a multifunctional organ.

Numerous mathematical models describing the airway geometry have emerged from analyses of morphometric measurements^2,5^. Weibel model A^2^ is one such simple but widely used model which assumes symmetric branching and 23 complete generations. However, in reality, bronchial trees in human lungs are neither symmetric nor regular and seldom complete. Raabe et al.^5^ reported measurements of airway diameters of four species: human, dog, rat, hamster. Several analyses^3,6^ of that data have yielded two conclusions—(i) the air flow rate and diameter of bronchioles within human lungs are related to each other by Hess–Murray law^7,8^ and (ii) the ratios of diameters of the major and minor daughters to their parent (homothety ratios) were found to be constants independent of generation (regular branching asymmetry). Hess–Murray law arises out of the optimization of the work required to maintain the flow and volume occupied by branched networks. Adherence to this law has been observed in vascular networks, xylem in plants as well as respiratory systems. Majumdar et al.^3^ also identified that such a regular branching asymmetry describes the distribution of diameters of bronchioles within every generation to a great extent. The fluid mechanics research community has discussed multiple investigations of an optimal bronchial tree geometry^9–11^, primarily based on flow resistance. Mauroy et al.^12^ argued that the overall flow resistance offered by an optimally designed bronchial tree dramatically escalates with bronchoconstriction but a shift from optimality as well as symmetry reduced that sensitivity. Using experimental evidence from PET scans and a simple computational model of a symmetric bronchial tree, Venegas et al.^13^ showed that bronchoconstriction leads to patchiness in lung ventilation and argued that the presence of even minimal heterogeneity leads to large clusters of poorly ventilated lung units, which might lead to failure of the respiratory system during an acute asthma attack. Along similar lines, Donovan^14,15^ demonstrated the role of structural and dynamic inhomogeneities on ventilation patterns in asthmatic and healthy subjects through computational modeling of clustered ventilation defect formation in bronchial trees. Through randomly generated constrictions, they found that a greater fraction of bronchioles are under-ventilated in asthmatic lungs in comparison to the control lungs. Florens et al.^16^ showed that asymmetry reduced the average oxygenation time while making its distribution more robust against stochastic variations. They also found that natural asymmetry of human airways is at a threshold limit, above which, oxygenation times become inadequate. However, the list of pragmatic needs of asymmetric branching is not complete without a discussion of its effect on one of the lung’s main functions—specifically, particle deposition in the tracheobronchial tree (to serve air purification).

The primary function served by the lung, i.e., gas exchange takes place only in the distal airways known as the acinus. Evolutionary design of these peripheral gas exchange units have been investigated by several authors^17,18^. In a review by Suki et al.^19^, the effects of mechanical forces on the physiological functioning of the lung parenchyma were outlined with a particular emphasis on the role played by collagen. The study elucidated the ways in which composition and intricate structure of the connective tissue influences the mechanical properties of the parenchyma. As Weibel et al.^18^ mentioned, the optimal airway tree design demands favouring convection in the central and diffusion in the peripheral airways. Also, alveolar permeability plays an important role with regards to diffusional screening and arrangement of alveoli in the deeper airways. In contrast to this body of literature, the main focus of the present study is on the structure and the convective function of the airways. In a recent and comprehensive review, Neelakantan et al.^20^ enlists multiple areas of scientific advancement (lung biophysics, biomechanics, physiology and medical imaging) which are integrated in computational modelling of the respiratory system and the manner by which these advances can benefit individualized diagnosis, prognosis and therapy evaluation in lung diseases.

In spite of studies on asymmetric bifurcations being reported back since 1976^5^, no study has discussed the role of lung asymmetry on functionality and performance. In this context, functionality of a lung can be characterized along multiple dimensions; we choose four—(i) total lung surface area, to be maximized for gas exchange, (ii) flow resistance, to be minimized, (iii) airway volume, to be minimized and (iv) particle deposition in the tracheobronchial tree, to be maximized (to ensure that clean filtered air reaches the acinus). The present study attempts to construct realistic geometric models of bronchial trees to study the effects of such asymmetry on this multidimensional lung function.

## Results

The structure and consequently the performance parameters of the bronchial tree are dependent on the degree of asymmetry (*r*) and cut-off diameter ($D_c$). $r=0.5$ represents symmetry and approaches 0 with increasing asymmetry. We study the effects of asymmetry on the number of terminal branches ($n_{tb}$), the total fluidic resistance to breathing ($\rho$), total lung volume (*V*) as well as the total particle deposition in the tracheobronchial tree ($\chi$).

### Number of terminal branches

**Figure 1:**
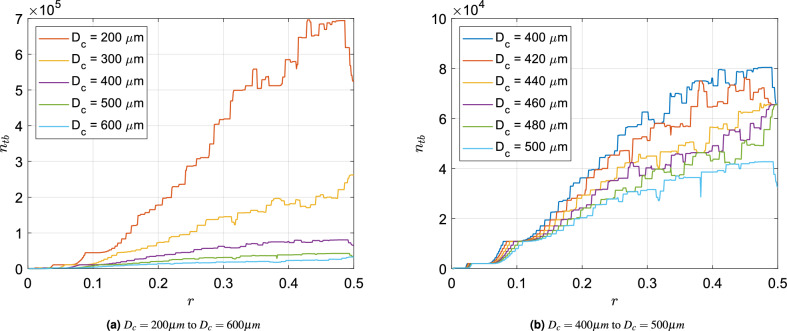
Effect of *r* on the number of terminal branches ($n_{tb}$) for different ranges of $D_c$—(**a**) $D_c = 200 \,{\upmu }\textrm{m}$ to $D_c = 600 \,{\upmu }\textrm{m}$ in steps of $100 \,{\upmu }\textrm{m}$ (**b**) $D_c = 400 \,{\upmu }\textrm{m}$ to $D_c = 500 \,{\upmu }\textrm{m}$ in steps of $20 \,{\upmu }\textrm{m}$. Maxima of all curves are either at $r=0.5$ or between $r=0.45$ and 0.5.

One of the most important geometric features of a bronchial tree is the number of branches at its distal end (in real lungs, these are the alveolar ducts terminating into alveolar sacs). The total surface area available for gas exchange is correlated to the number of terminal branches and is expected to be maximised in an optimal lung. Figure 1a,b show the variation of number of terminal branches ($n_{tb}$) with *r* for different ranges of $D_c$. The variations are non-monotonic and contain abrupt jumps. The non-monotonicity arises from the fact that cutoff is a binary logical operation that results in the presence/absence of a bronchiole along with the sub-tree originating from it. This non-linearity compounded over several generations is the essential source of this interesting behaviour. For a detailed explanation of the origins of this non-monotonicity, the reader is referred to the “Methods” section.

For symmetric branching ($r=0.5$), the *N*th generation is the terminal generation if the diameter of the $(N+1)$th generation is less than $D_c$. Consequently, the total number of terminal branches is $2^{N}$, all belonging to the *N*th generation. Figure 1b shows that the curves converge at certain points at $r=0.5$. Those points refer to $n_{tb} = 2^N$ such that $\left( \frac{1}{\root 3 \of {2}}\right) ^N D_0> D_c > \left( \frac{1}{\root 3 \of {2}}\right) ^{N+1} D_0$ (for $r=0.5$, $\kappa _{maj}=\kappa _{min}=\frac{1}{\root 3 \of {2}}$).

As *r* decreases from the symmetric value of 0.5, interesting effects come into play and the curves diverge. Due to increasing asymmetry, major daughter branches get larger in size and minor ones become even smaller. For cases where $D_c$ is just above $\left( \frac{1}{\root 3 \of {2}}\right) ^{(N+1)} D_0$, $n_{tb}$ increases with decreasing *r*. This is because as asymmetry sets in, an increasing number of larger branches from the $(N+1)$th generation are added to the bronchial tree than the smaller ones which are subtracted from the *N*th generation. For example, let us consider the case with $D_c = 420 \,{\upmu }\textrm{m}$. Here, $N=16$, $n_{tb} = 2^{16} =65536$ at $r=0.5$ and $\left( \frac{1}{\root 3 \of {2}}\right) ^{N} D_0 = 496 \,{\upmu }\textrm{m}$. As *r* is reduced, $n_{tb}$ increases and reaches a maxima of 76305 at $r=0.450$.

Figure 1b shows a finegrained variation of $D_c$. It can be observed that there is a gradual change in the curves with increasing $D_c$. The gross slope of the curve at $r=0.5$ changes from negative to positive with increasing $D_c$. There is a shift from having a maximum at an intermediate value of *r* to having one at $r=0.5$. Therefore, we can conclude that $D_c$ plays an important role in determining optimal *r* value where $n_{tb}$ exhibits a maximum. In other words, there exists an optimum value of *r* for which $n_{tb}$ is maximised. Thus, a small but finite asymmetry could be nature’s way of maximising the total surface area available for gas exchange. However, the general trend of these irregular curves suggests that as *r* goes from 0 to 0.5, the number of terminal branches (hence, the surface area available for gas exchange) increases. With this general trend as the background, one can however observe the sharp changes in $n_{tb}$ with *r* or $D_c$, pointing to a possible source of significant inter-subject variability, which has been a subject of recent interest in the literature^21^.

### Resistance

**Figure 2:**
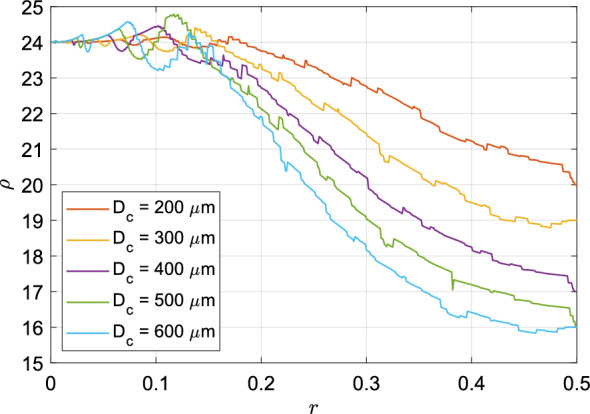
Effect of *r* on the normalised fluidic resistance ($\rho$) offered by the entire tracheobronchial tree for different values of $D_c$. Low values of *r* cause unphysical fluctuations in the resistance. Minima of all curves are either at $r=0.5$ or between $r=0.45$ and 0.5.

For achieving proper ventilation, air has to be inhaled and exhaled during each breathing cycle. Inhalation is an active process executed by a group of muscles (diaphragm, external intercostal muscles) which increase the volume of the thoracic cavity. Exhalation, at rest, is a passive process. However, during exercise or strenuous activity, exhalation can also be actively achieved through effort of several muscles (internal intercostal muscles, abdominal muscles). All in all, there is a significant energy expenditure on the vitally necessary act of breathing. There are two components of the “Work of breathing”—compliance work (which accounts for approximately $65 \%$ of the total work) and fluidic resistance work (approximately $35 \%$). Compliance work refers to the work of expanding the lung and the thorax, both of which are viscoelastic bodies. Resistance work refers to the work required in overcoming viscous dissipation in the flow of air through the lung airways. The structure of the bronchial tree dictates the flow resistance offered by it, which should be minimised.

For the purpose of this model, it is assumed that the mouth of the trachea is at atmospheric pressure and the pressure inside all the terminal branches are the same and equal to the intra-alveolar pressure. We also assume that compliance work is unaffected by airway geometry. Effectively, the lung is a network of resistances connected in parallel. The equivalent resistance of such a bronchial tree is calculated using an electrical circuit analogy, with resistance of each bronchiole ($R_i$) given by the Hagen-Poiseuille equation, shown in Eq. (1).

1 $$\begin{aligned} R_i = \frac{\Delta p}{Q} = \frac{128\mu L_i}{\pi D_i^4} \end{aligned}$$

Here $L_i$ and $D_i$ are the length and diameter of the *i*th bronchiole, respectively. A dimensionless resistance of the bronchial tree $(\rho )$ can be defined as the ratio of resistance of the entire bronchial tree to that of the trachea. Figure 2 shows the variation of $\rho$ with *r* for different values of $D_c$. The general trend in this figure is that the equivalent resistance decreases as *r* approaches 0.5. With the exception of a few cases, the minima of theses curves were observed at $r=0.5$. An important point to note here is that the differences in resistance offered by bronchial trees arise by virtue of incompleteness of the trees only. Had the bronchial trees been complete ($D_c = 0$), the overall resistance ratio for all values of *r* would be the same and equal to 24. This is a consequence of following Hess–Murray law in determining diameters of bronchioles, which ensures that resistance offered by each generation in a dichotomously branching tree is equal for any level of asymmetry (hence, for 23 generations, $\rho = 24$ $\forall$ *r*).

### Volume

**Figure 3:**
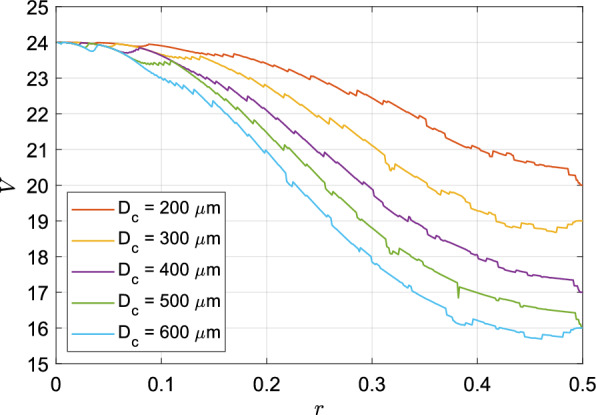
Effect of *r* on the dimensionless tracheobronchial tree volume (*V*) for different values of $D_c$. Minima of all curves are either at $r=0.5$ or between $r=0.45$ and 0.5.

The lungs occupy a sizable portion of the thoracic cavity and share the available space with several other organs. Therefore, the optimal structure of the lungs must ensure functional efficiency while minimising occupied volume. The total volume of the bronchial tree is calculated by adding the volumes of all the bronchioles (assumed to be straight circular cylinders). The ratio of this volume of the entire bronchial tree to that of the trachea is defined as the dimensionless airway volume (*V*). Figure 3 shows the variation of *V* versus *r* for different values of $D_c$. It is observed that *V* is minimised as *r* approaches 0.5 (barring a few exceptions). With increasing asymmetry (decreasing *r*), a higher number of major daughters (along with attached sub-trees) penetrate into the deeper generations, increasing the volume of the entire network.

### Tracheobronchial particle deposition

Apart from gas exchange, air filtering is an important function served by the lungs. The lungs provide protection against inhaled foreign particles and airborne pathogens. As particle-laden air passes through the airways, the particles are deposited on the mucous-lined walls of the bronchioles due to mechanisms of inertial impaction, Brownian diffusion and gravitational settling^21–28^. The pathogens are then neutralised by T-cells and by the immunological system. The mucosa is then transported towards the nasal passage by ciliary action. Particle deposition is heavily dependent on the structure of the airways. In a recent work, Christou et al.^29^ have assessed the breadth of anatomical variability of the upper airways in general populations and its probable impact on aerosol deposition in the lungs. More work is needed to fully comprehend inter-subject variability in morphometry.

Tracheobronchial particle deposition was calculated using a multi-path deposition model. We will briefly describe this particle deposition model. The extra-thoracic deposition was calculated using the empirical correlations from Cheng et al.^30^. From their study, the deposition efficiency in the extra-thoracic region for either nasal or oral breathing is expressed as:

2a $$\begin{aligned} \eta _n&= 1 - e^{(-0.00309 d_p^2 - 16.6 D_b^{0.50}Q^{-0.28})} \end{aligned}$$

2b $$\begin{aligned} \eta _o&= 1 - e^{(-0.000278 d_p^2 - 20.4 D_b^{0.66}Q^{-0.31})} \end{aligned}$$

where, $d_p$ is the particle diameter, *Q* is the volumetric flow rate. Particle diffusion by Brownian motion is calculated using the diffusion coefficient $D_b$ expressed by $D_b = \frac{C_s k T}{3 \pi \mu d_p}$, where *k* is the Boltzmann constant, *T* is the absolute temperature and $\mu$ is the dynamic viscosity of air. The Cunningham slip correction factor^31^ is given by $C_s = 1 + (2 l/d_p)[A_1 + A_2 e^{-A_3 d_p/l}]$, where $A_1=1.257$, $A_2=0.4$ and $A_3=0.55$ are empirically determined constants^32,33^. For air at sea level, mean free path length *l* is approximately 70 nm.

Particles entering the bronchial tree are deposited on the walls of the bronchioles through three major mechanisms—(i) inertial impaction (ii) sedimentation and (iii) diffusion. There are multiple analytical expressions published in literature to estimate particle deposition efficiency through each physical mechanism. Reviews by Hofmann^23,24^ and Darquenne^25^ enlist a number of such models.

**Figure 4:**
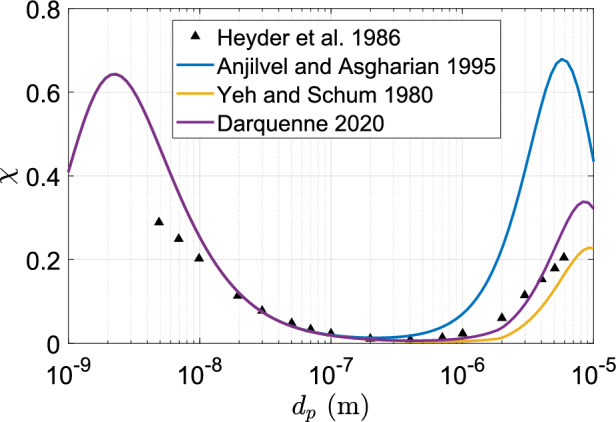
Tracheobronchial particle deposition fraction ($\chi$) as a function of particle diameter ($d_p$), as predicted by different deposition models for specified oral breathing conditions (Tidal volume = 1000 ml, Breathing frequency = $15\,\textrm{min}^{-1}$). The morphometric parameters considered for lung geometry were $r = 0.5$ (symmetric) and $D_c = 500 \,{\upmu }\textrm{m}$.

The choice of the bronchial tree particle deposition model is important. In order to objectively choose the appropriate model, we have compared the predictions of various models available in literature due to Yeh and Schum^26^, Anjilvel and Asgharian^28^ and Darquenne^25^) with the corrected experimental data from Heyder et al.^34^. Figure 4 shows the tracheobronchial deposition fraction ($\chi$) as a function of particle diameter ($d_p$), as predicted by the different deposition models. From this figure, it is apparent that the most recent model due to Darquenne^25^ yields the best comparison. We have hence chosen that model for the current work and is described next.

The particle deposition in each bronchiole is known to occur through impaction, sedimentation and diffusion. The particle deposition efficiencies for the respective mechanisms ($\eta _i$, $\eta _s$ and $\eta _d$) are given by:

3a $$\begin{aligned} \eta _i&= 1.3(Stk - 0.001) ~~ \text { for } ~ Stk \ge 0.001 \end{aligned}$$

3b $$\begin{aligned} \eta _s&= 1 - e^{-\frac{4 C_s v_s L}{\pi u D}} \end{aligned}$$

3c $$\begin{aligned} \eta _d&= 1 - 0.819e^{-7.315 \Delta } - 0.0976 e^{-44.61 \Delta } - 0.0325 e^{-114 \Delta } - 0.0509 e^{-79.31 \Delta ^{2/3}} \end{aligned}$$

Here, Stokes number $Stk = \frac{C_s \rho _p d_p^2 u }{18 \mu D}$, terminal settling velocity $v_s = \frac{\rho _p d_p^2 g }{18 \mu }$ and diffusion parameter $\Delta = \frac{D_b L}{u D^2}$. $\rho _p$ is the particle density and taken as $1000\, \textrm{kg}/\textrm{m}^3$ and *g* is the acceleration due to gravity. Here, *L*, *D* and *u* are respectively the length, diameter and flow velocity corresponding to each individual bronchiole. The mean diameter of the particles $d_p$ was taken as $10 \,{\upmu }\textrm{m}$ (characteristic drop sizes generated during loud speech^35^). The kinematic viscosity ($\mu$) of air was taken as $1.5\times 10^{-5} \textrm{m}^2 /\textrm{s}$.

The deposition efficiency ($\eta$) in each bronchiole was determined by superposition of all three mechanisms.

4 $$\begin{aligned} \eta = \eta _i + \eta _s + \eta _d \end{aligned}$$

The flow division through the bronchial tree and the deposition efficiencies of all bronchioles were determined for a 2 s inhalation of 500 ml of air (average $Q = 2.5 \times 10^{-4}\, \textrm{m}^3 /\textrm{s}$). Then, particle propagation and deposition through the bronchial tree was simulated. The fraction of particles deposited in each bronchiole ($\chi _b$) was calculated as the product of the number of particles entering a bronchiole and its deposition efficiency ($\eta$). Finally, the total fraction of inhaled particles ($\chi$) which were deposited in the non-terminal branches was calculated as the summation of fractions over all bronchioles in the tracheobronchial tree. This fraction is a measure of the protection that the lung offers against pathogen transport into the deep lung. The greater the value of $\chi$, the greater the protection against the acinus being exposed to pathogens. On a related note, a series of experimental studies by Kim et al.^36–38^ have conclusively demonstrated that branching angle does not have a significant effect on particle deposition efficiency unless it is unphysically high ($60^{\circ }$ to $90^{\circ }$). In human lungs, this branching angle is typically less than or equal to $45^{\circ }$^26^. Hence, we have not modeled the effects of branching angle in our study.

This novel framework developed for modelling the deposition of inhaled particles within the bronchial tree has a number of applications. These calculations are of physiological importance in multiple ways. It also paints a picture of distributed patterns of deposited particles within the lung. Viral load can be estimated in case of airborne disease transmission. For targeted drug delivery through inhalation, patient specific drug load can be determined if the morphometric parameters are known.^39,40^ This method of studying a tracheobronchial tree complements CFD approaches which can currently handle up to about ten generations^41^. Using this reduced order modeling approach, one can functionally study a complete tracheobronchial tree.

**Figure 5:**
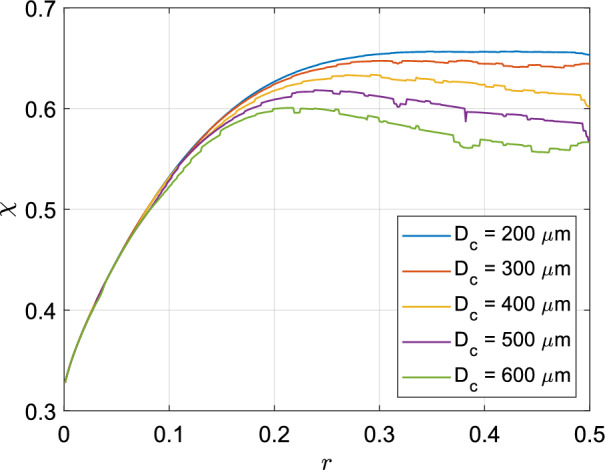
Effect of *r* on tracheobronchial particle deposition fraction ($\chi$) for different values of $D_c$. It can be observed that as *r* is reduced from 0.5, the deposition fraction increases to reach a maxima in the intermediate values of *r* and then decreases to approach its minima as $r \rightarrow 0$.

Figure 5 shows the variation of the overall deposition fraction ($\chi$) as a function of flow dividing ratio *r* for different values of $D_c$. It can be observed that with decreasing *r* (increase in degree of asymmetry), $\chi$ increases, reaches a maximum at an intermediate value of *r*, again decreases and approaches its minima as $r \rightarrow 0$. From this figure, it is clear that asymmetry ($r \ne 0.5$) is required to maximise tracheobronchial particle deposition and to offer the highest protection against foreign particles. The reasons behind these observations are discussed next.

**Figure 6:**
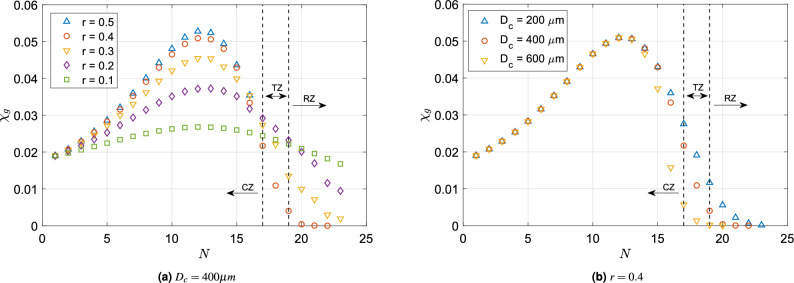
Particle deposition fractions in each generation ($\chi _g$) versus generation number (*N*) for (**a**) different values of *r* with fixed value of $D_c = 400 \,{\upmu }\textrm{m}$ and for (**b**) different values of $D_c$ with fixed value of $r = 0.4$. Deposition in terminal branches of each generation was excluded during the calculations. These figures show the individual effects which *r* and $D_c$ have on the manner in which particle deposition is distributed across generations. Generations upto the 16th generation refer to the conducting zone (CZ), those from the 17th to the 19th generation are the transitional zone (TZ) and from 20th generation onward belongs to the respiratory zone (RZ).

Figure 6a shows the total fraction of inhaled particles deposited in each generation ($\chi _g$) for five different values of *r* (0.1 to 0.5) corresponding to a fixed value of $D_c$ ($= 400 \,{\upmu }\textrm{m}$). It can be observed that the deposition fractions of each generation ($\chi _g$) reduce with decreasing *r* in the upper generations (conducting zone). There is a crossover point in the transitional zone. From the 17th generation onward, $\chi _g$ increases with decreasing *r*. Figure 6b shows the fraction of inhaled particles deposited ($\chi _g$) in each generation for three cases as a function of the cutoff diameter, $D_c$ for $r = 0.4$. It can be observed here that the deposition in each generation is the same for the three different cases up to the 13th generation. With an increase in cut-off diameter $D_c$, the number of bronchioles in the lower generations decreases, which leads to a decrease in the total tracheobronchial particle deposition. Also, the particle deposition fraction is greatest in the 10th to 15th generations. The reasons underlying this observation—the fact that $\chi _g$ increases and then decreases with increasing *N*—can be explained by investigating the distributions of bronchiole diameter and deposition efficiencies within a particular generation.

**Figure 7:**
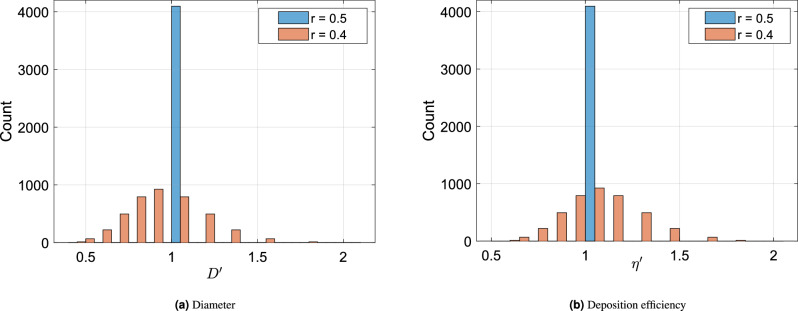
Histograms of normalized (**a**) bronchiole diameter ($D'$) and (**b**) deposition efficiency ($\eta '$) in bronchioles of the 12th generation. A symmetric lung ($r = 0.5$) shows a Dirac-delta distribution of diameters and therefore, particle deposition. For $r=0.4$, the diameters follow a binomial distribution and the deposition efficiency distribution shows a long tail. All variables have been normalized against their values for the symmetric case ($r=0.5$) as $x' = \frac{x-x_{0.5}}{x_{0.5}}$.

Figure 7 shows the histograms of normalized brochiole diameters and normalized deposition efficiencies (normalized with respect to the values for the symmetric case) of bronchioles of a representative 12th generation for two values of *r*. For $r=0.5$, all bronchioles have the same diameter (symmetric branching) and as a consequence, the same $\eta$ for all bronchioles; the distributions are Dirac-delta functions. For $r=0.4$ (taken as an example), the bronchiole diameters follow a binomial distribution with a small number of both large and small diameter bronchioles (see Fig. 7a). It must be noted that the mode of the distribution for $r=0.4$ has shifted to a diameter less than 1. In other words, as *r* decreases, the mode of the distribution will also decrease, while the width of the distribution will continue to increase. The arithmetic mean of the diameters at a particular generation is also lower than the value for a symmetric tree. This is due to the nonlinearity associated with the Hess–Murray law. Correspondingly, these smaller bronchioles give rise to higher deposition efficiencies as is evident from the long tailed distribution of $\eta$, as shown in Fig. 7b.

**Figure 8:**
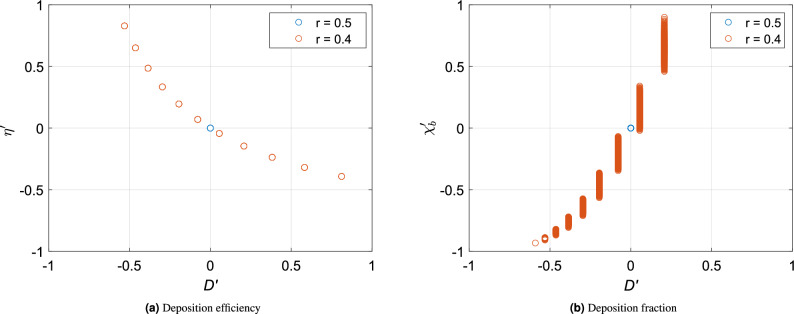
Scatter plots of normalized (**a**) deposition efficiencies ($\eta '$) and (**b**) bronchial particle deposition fractions ($\chi _b'$) against the normalized bronchial diameters ($D'$) of the 12th generation in two bronchial trees (with $D_c = 400 \,{\upmu }\textrm{m}$)—one symmetric ($r = 0.5$) and the other asymmetric ($r = 0.4$). All variables have been normalized against their values for the symmetric case ($r=0.5$) as $x' = \frac{x-x_{0.5}}{x_{0.5}}$.

A cursory analysis of this result could indicate that increased asymmetry should result in a higher deposition in a particular generation, due to the presence of smaller bronchioles with increasing asymmetry. However, that is not the complete story. The smaller bronchioles are indeed effective filters, but the number of particles that they receive is also lower. A smaller bronchiole is likely to arise from a smaller predecessor parent in the earlier generation which is also a relatively effective filter. In addition, a smaller daughter bronchiole is also likely to see a lower flow rate in comparison to the other (major) daughter (due to Hess–Murray law). We illustrate this point with Fig. 8, which are plots of normalized deposition efficiency ($\eta '$) and normalized deposition fraction ($\chi '_b$) versus normalized bronchiole diameter ($D'$). The values have been normalized as deviation from the values for a symmetric tracheobronchial tree ($r=0.5$). It can be observed from Fig. 8a that the normalized deposition efficiency ($\eta '$) decreases with increasing diameter. However, the actual deposition fraction ($\chi _b'$) is higher for bronchioles of bigger diameter. This is a product of an increasing function—concentration of particles entering the bronchiole of a given generation—and a decreasing function—the particle deposition efficiency of those bronchioles. This product is lower for smaller bronchioles. As a result, the actual deposition fraction in these bronchioles ($\chi _b$) is lower in spite of having a higher deposition efficiency. A corroboration of this argument is presented in Fig. 8b which is a plot of the normalized bronchiole deposition fraction ($\chi _b'$) versus normalized bronchiole diameter ($D'$) at the 12th generation in a bronchial tree with $r=0.4$. With an increase in asymmetry, the reduction in deposition in the smaller branches of a generation is greater than the corresponding increase in the larger branches. As a result, the deposition fraction at any generation ($\chi _g$) in the upper generations reduces with decreasing *r*. The same effect explains why $\chi _g$ initially increases and then decreases with *N*.

This effect also drives the initial increase and then the decrease in total tracheobronchial deposition $\chi$ as *r* is reduced from 0.5. Moreover, another related effect of asymmetry is the initial introduction of additional major branches in the lower generations, where more particles are likely to be deposited before they reach the acinus. However, at high levels of asymmetry, the number of these branches decreases because minor branches (and attached sub-trees) get terminated. The initial increase in tracheobronchial deposition ($\chi$) with decreasing *r* is the result of two effects of asymmetry on the lower generations—(i) increase in generational deposition fraction ($\chi _g$) and (ii) increase in number of branches. The subsequent decrease in $\chi$ is again the result of two concomitant effects—(i) decrease in generational deposition fraction ($\chi _g$) in the upper generations and (ii) decreasing number of branches in the lower generations (which would have had a relatively higher deposition efficiency, $\eta$). This causes the overall deposition to initially increase, reach a maximum and then decrease with asymmetry (see Fig. 5).

## Discussion

The research question that we ask is, why is asymmetric bronchial bifurcation, a feature of the lung? To answer that question, we investigate the connection between the degree of branching asymmetry to several lung performance parameters. We observe that maximum surface area, minimum resistance and minimum volume are obtained either exactly at $r=0.5$ (symmetry) or in its neighborhood, indicating that a symmetrically branched tracheobronchial tree optimizes those performance functions.

For $D_c = 400\, \upmu \textrm{m}$, $\chi$ is maximised at $r = 0.292$. This value is less than $10\%$ lower than the value measured in human lungs^3^. In addition, the maximum value of $\chi$ is $5.2 \%$ greater than its value at $r=0.5$. If *r* is reduced from 0.5 to 0.326 (the value of *r* observed in human lungs^3^), $\chi$ increases by $4.4 \%$ whereas $n_{tb}$ reduces by $11.35\%$, $\rho$ increases by $14.47\%$ and *V* increases by $13.07\%$. Therefore, one can see how natural asymmetry ($r\approx 0.326$)^3^ enhances protection at the cost of performance on other functions.

Counter-intuitively, the lung has evolved to emphasise protection against pathogens and toxins far more than maximizing either lung surface area (for gas transport) or to minimise fluid mechanic resistance to breathing or breathing work. After all, the lung is the most exposed vital organ in the whole body unlike heart, brain, etc. Lungs come in direct contact with the external atmosphere and more importantly, exposes blood (via pulmonary circulation) to the dangerous airborne pathogens. Therefore, nature emphasises risk aversion first, then efficient performance. There is no point in optimizing the lung if its host does not live a long, healthy life.

This work is an initial step towards more systematic studies on bronchial asymmetry since there are several assumptions and idealizations that were necessary to achieve the model. Lung geometry was estimated using simple deterministic equations. Particle deposition was calculated using experimentally-validated analytical expressions to model the different physical mechanisms.

**Figure 9:**
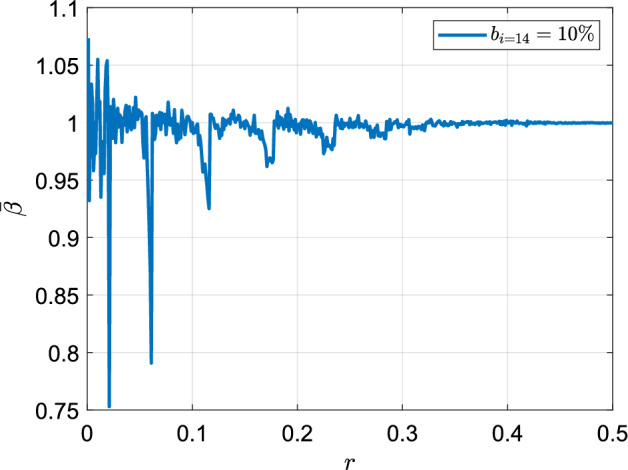
Variation of $\bar{\beta }$ as a function of *r*. It can be observed that *r* does not have a significant effect on $\bar{\beta }$ which lies close to 1. The are exceptions at low values of *r*, which represent cases of extreme asymmetry.

A potential application use case of the work presented in this manuscript is to understand the acinar ramifications of diseased lung condition as well as inter-subject variability. Due to various pathophysiological conditions, there are ventilation heterogeneities across different regions in the airways^13–15^. Sometimes such heterogeneity may occur in healthy individuals as well in that all airways are not recruited evenly. This study, which assumes all paths of the airways are recruited can be thought of as an upper limit of the capacity of human lungs. However, from a modelling perspective, it would be interesting to study the performance degradation (or conversely the robustness of lung design) when a fraction of the airways are constricted. Towards this end, a fraction of the airways ($\sim 10 \%$ in the 14th generation) were randomly constricted to simulate the effect of ventilation heterogeneity. The impact of this blockage on the ventilated surface area was quantified using $\beta$, defined as the ratio of the relative change in the number of terminal branches that were ventilated $(\frac{\Delta n_{tb}}{n_{tb}})$ to the relative change in the number of bronchioles $(\frac{\Delta b}{b})$ in the specific generation. Figure 9 shows the mean value of $\beta$ (denoted as $\bar{\beta }$, obtained as the mean of 1000 realizations of Monte Carlo simulations) as a function of *r*. The degree of asymmetry did not significantly affect $\bar{\beta }$ $(\approx 1)$ unless the asymmetry was extreme $(r < 0.1)$. When *r* was low, the deviation of $\bar{\beta }$ from 1 was large due to the wide variation in sub-trees attached to bronchioles of any generation. Some bronchioles were attached to very large sub-trees, while others were attached to smaller sub-trees, leading to random blockages either causing a blockage of a large number of bronchioles in the larger sub-tree attached to it (resulting in $\bar{\beta } > 1$) or causing no significant change ($\bar{\beta } < 1$). The number of bronchioles attached to large sub-trees in the range of $0< r < 0.1$ (which are anyway unphysical) was statistically less, which explained the sharp drops in $\bar{\beta }$. More nuances can be added by including medically viable assumptions on which pathways are more likely to be constricted, instead of treating all pathways as being equally susceptible. To adapt our deposition model, we could modify the model parameters to account for changes in ventilation heterogeneities. For example, the deposition efficiency could be adjusted based on the regional distribution of ventilation or the presence of airflow constrictions in certain regions of the lungs. On the topic of inter-subject variability, Islam et al.^42^ remark in their review of pulmonary drug delivery procedures about the huge inter-patient variability of drug dosage delivered to the deep lungs being a major concern. Modeling efforts like the present study will be a key factor in developing efficient and personalised drug delivery systems in the future^39,40^.

## Methods

**Figure 10:**
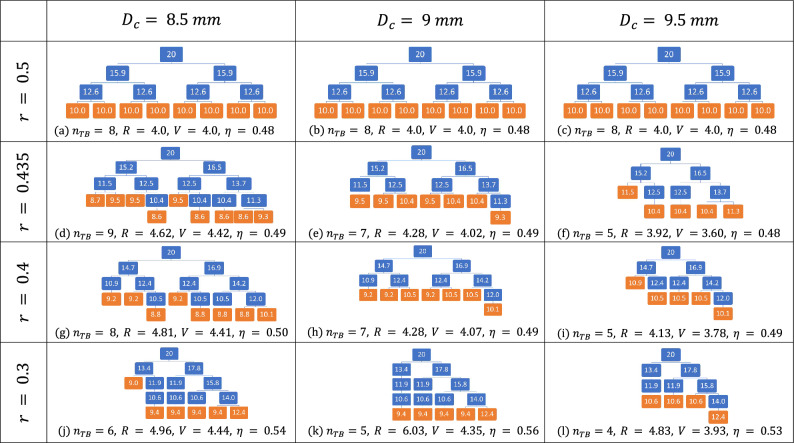
Illustrative examples outlining the effects of *r* and $D_c$ on bronchial tree geometry, each small block represents a bronchiole with its diameter (in mm) written on it. As shown in (**a**–**c**), for $r=0.5$, the bronchial trees are symmetric (same for all three values of $D_c$) but terminated at the 3rd generation only and has 8 terminal branches (in orange). Had $D_c$ been equal to zero, the complete tree would have 16 terminal branches in the 4th generation. For $D_c=8.5\,\textrm{mm}$, $n_{tb}=9$ is at its maxima at $r=0.435$. (**d**) A few branches penetrating into the 4th generation. However for $D_c = 9$ and $9.5\,\textrm{mm}$, the previously present bronchioles are now absent as their diameter would fall below $D_c$, depicted in (**e**, **f**), respectively. This is what causes them to have $n_{tb} = 7$ and 5, respectively (lower than $n_{tb} = 8$ at $r=0.5$). The effects of further reduction of *r* to 0.4 and 0.3 can be observed in the changes going from (**g** to **j**, **h** to **k** and **i** to **l**), respectively.

**Figure 11:**
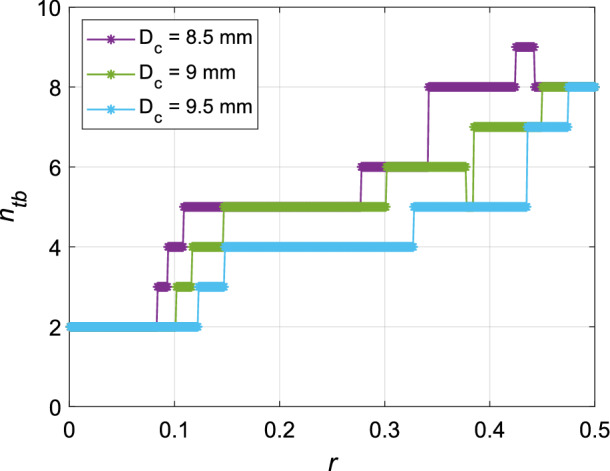
Number of terminal bronchioles of a five generation model lung as a function of *r* for $D_c = 8.5, 9, 9.5 \textrm{mm}$.

A bronchial tree is mathematically modelled as successively, dichotomously and asymmetrically branched network. The *i*th generation (with the trachea as the 0th generation) was represented by a row matrix with $2^{i}$ elements, where the *j*th element represented the *j*th bronchiole of the *i*th generation. The diameter of the trachea was taken as 20 mm (consistent with typical morphometric measurements^2,5^). Each parent branch bifurcates into two daughter branches—major and minor—of unequal diameters (in the most general case). The diameters of the subsequent branches were generated based on the rules discussed hereunder. The airways were assumed to follow Hess–Murray law^7,8^, which relates the volumetric flow rate ($Q_{i,j}$) through the *j*th bronchiole of the *i*th generation with its diameter ($D_{i,j}$) and is given by

5 $$\begin{aligned} Q_{i,j} = C D_{i,j}^n \end{aligned}$$

*C* and *n* (flow coefficient $n = 3$ for laminar flows and has been assumed to be the case in this work, arising from Hess–Murray law) are constants. Volumetric air flow rate through a parent bronchiole, $Q_{ij}$ divides into $Q_{i+1,2j}$ and $Q_{i+1,2j+1}$ between its minor and major daughter bronchioles, respectively. Hence, from continuity of incompressible flow,

6 $$\begin{aligned} Q_{i,j} = Q_{i+1,2j} + Q_{i+1,2j+1} \end{aligned}$$

For symmetric branching, $Q_{i+1,2j} = Q_{i+1,2j+1} = Q_{i,j}/2$ implying $r=0.5$. But in case of asymmetric branching, the minor daughter carries lesser volume flow rate than the major daughter. In this case, the flow dividing ratio *r*, becomes an important parameter in quantifying the degree of branching asymmetry and is defined as,

7 $$\begin{aligned} r = \frac{Q_{i+1,2j}}{Q_{i,j}} \left( 0 < r \le 0.5 \right) \end{aligned}$$

From Eqs. (5), (6) and (7), diameters of the daughter bronchioles can be determined as,

8 $$\begin{aligned} D_{i+1,2j} = D_{i,j} r^{1/n} \quad D_{i+1,2j+1} = D_{i,j} (1-r)^{1/n} \end{aligned}$$

where $D_{i,j}$ is the diameter of the parent bronchiole and the minor and major daughters have diameters $D_{i+1,2j}$ and $D_{i+1,2j+1}$, respectively. From the literature^3^, it can be learnt that the asymmetry at each branching is *regular*, i.e, the homothety ratios $\kappa _{minor} = {D_{i+1,2j}}/{D_{i,j}}$ & $\kappa _{major} = {D_{i+1,2j+1}}/{D_{i,j}}$ remain constant, independent of the generation. As a result, the flow dividing ratio, *r* is also constant for all generations of the bronchial tree.

One flaw in assuming bronchial trees to be complete ($2^n$ bronchioles present in all *n* generations) is that for asymmetric cases, many bronchioles in the distal generations are assigned unrealistic diameters (typically smaller than typical alveoli). Such diameters are not realistically possible as there are biological limits to how small terminal branches can be. As a correction, the concept of cut-off diameter ($D_c$) has been introduced as has been proposed previously^3,43^. Starting from the trachea, diameters of daughter bronchioles of the subsequent generations are determined and once any bronchiole diameter becomes smaller than the cut-off diameter ($D_c$), that bronchiole is deemed to be the terminal branch. The geometries of the generated bronchial trees are determined by two parameters, viz., degree of asymmetry (*r*) and the cut-off diameter ($D_c$) and the effects of those parameters on lung performance was investigated. Multiple such bronchial trees were generated for *r* ranging from 0 to 0.5, in steps of 0.001, while keeping $D_c$ constant. Then, $D_c$ is changed from 200 to $600\,\upmu \textrm{m}$, in steps of $100\,\upmu \textrm{m}$ in order to observe the effect of $D_c$.

To illustrate the dependence of the geometry of the trees on *r* and $D_c$, let us consider an example tree limited to 5 generations (trachea and 4 subsequent generations). The value of *r* is varied from 0 to 0.5 while varying the cut-off diameter over three different values of $D_c = 8.5$, 9 and 9.5 mm. Figure 10 shows the morphology as well as calculated diameters of the bronchioles for these values of *r* and $D_c$. Figure 11 shows the variation of number of terminal branches $n_{tb}$ with respect to *r* for constant $D_c$ (obtained by a finer grained variation of *r* in comparison to Fig. 10). It is seen from Fig. 11 that for $D_c=8.5\,\textrm{mm}$, within $0.425 \le r \le 0.442$, $n_{tb}$ is maximised ($n_{tb} = 9$). For $D_c=9\, \textrm{mm}$, there is non-monotonic decay with reduction of *r* whereas for $D_c=9.5\,\textrm{mm}$, the trend is monotonic. Both of them have their maxima of $n_{tb} = 8$ at $r=0.5$ only. In general, as $D_c$ increases, $n_{tb}$ decreases because bronchioles of diameters smaller than $D_c$ are terminated. With increasing asymmetry (decreasing *r*), the balance between addition of newer branches penetrating to deeper generations and losing out branches in the upper generations (along with the sub-trees attached to them) dictate whether $n_{tb}$ would increase or decrease. In spite of being simplistic examples, these are illustrative of the nature of morphometry for full-scale 23-generation bronchial trees as well.

## Data Availability

The datasets used and/or analysed during the current study available from the corresponding author on reasonable request.
